# Genetic variant of *WIF1* gene is functionally associated with developmental dysplasia of the hip in Han Chinese population

**DOI:** 10.1038/s41598-018-36532-8

**Published:** 2019-01-22

**Authors:** Ye Sun, Yongqing You, Kerong Dai, Junxin Zhang, Moqi Yan, Yijian Zhang

**Affiliations:** 1 Shanghai Key Laboratory of Orthopaedic Implants, Department of Orthopaedic Surgery, Shanghai Ninth People’s Hospital, Shanghai Jiao Tong University School of Medicine, Shanghai, 200011 China; 2 Department of Nephrology, Affiliated Hospital of Nanjing Medical University, North District of Suzhou Municipal Hospital, Suzhou, China; 3 Department of Orthopedics, The First Affiliated Hospital of Soochow University, Suzhou, 215006 China

## Abstract

Developmental dysplasia of the hip (DDH) is a common skeletal disorder. Studies have demonstrated a significant role of *WIF1* gene in skeletal development. The present study was conducted to reveal the association between DDH and gene *WIF1*. A two-stage case-control candidate gene association study was conducted in total 1573 samples (586 DDH patients and 987 healthy controls) in this study. Polymorphism rs3782499 was genotyped in all samples. Difference of WIF1 expression in hip joint tissue was compared between the patients and the controls. WIF1 expression was compared among different genotypes in DDH patients. The SNP rs3782499 was found significantly associated with DDH in the two-stage study with 585 patients and 987 controls. There was a significant difference in allele frequency (p = 4.37 * 10^−5^) and genotype distribution in a recessive model (AG + GG vs. AA). DDH patients were found to have significantly higher WIF1 expression than controls. Moreover, Patients with rs3782499 genotype AA have a significantly increased expression of WIF1 than those with GG. To conclude, polymorphism rs3782499 of *WIF1* gene is a functional variant regulating the expression of WIF1 in DDH in Chinese Han population, which might be a potential biomarker for the early diagnosis of DDH.

## Introduction

Developmental dysplasia of the hip (DDH) (OMIM#142700) is a common disorder and a significant risk factor for hip osteoarthritis in adults. It is reported that the incidence of DDH is different worldwide, ranging from 0.1 to 10%^1^ DDH patients are characterized by laxity of hip joint and dysplasia in the hip, presenting as hip incongruence and secondary dislocation. Subsequent dysfunction leads to an unacceptable burden in bone development and physical development. DDH comprises a spectrum that include abnormal acetabular shape (dysplasia) and malposition of the femoral head, ranging from dislocated hip and mild subluxation to fixed dislocation, leading to permanent disabilities in later years if the diagnosis is delayed with the patient left untreated. Early diagnosis is most important to achieve successful clinical prognosis in the treatment of DDH. Previously published studies have suggested that breech positioning in delivery, a family history of DDH, first born, pes equinovarus (PEV), female gender, swaddling and large birth size are associated with DDH. Despite mechanical factors revealed in previous studies, DDH is considered a genetic disorder. Several DDH susceptibility genes (e.g. GDF5, TBX4, ASPN, PAPPA2, HSPG2, ATP2B4 and TGFB1) has discovered in multiple populations^2–7^. However, disease-causing variants have yet to be identified and the complex etiology of DDH has not been elucidated.

Wnt inhibitory factor 1 (WIF1) encoded by the WIF1 gene is a lipid-binding protein that binds to Wnt proteins. WIF1 is composed of a WNT inhibitory factor (WIF) domain and five epidermal growth factor (EGF)-like domains. WIF gene is a marker of osteogenesis and protein encoded by WIF1 inhibits WNT signal, which plays a significant role in bone and joint development of embryos. WIF1 has been demonstrated in the development of various bone malignant tumors (e.g. osteosarcoma, Ewing’s sarcoma and chondrosarcoma)^8^. WIF1 was also reported to be the causative gene of a Nail-Patella-like disorder^9^. Several lines of evidence suggest that WIF1 plays an important role in skeletal development. Nevertheless, there is no study of WIF1 concerning developmental dysplasia of the hip.

In this study, we explore the association between WIF1 gene and DDH in the Han Chinese population.

## Materials and Methods

### Patients

A two-stage method was applied in this study. In stage 1, we enrolled 386 radiologically confirmed DDH patients and 558 healthy controls. In stage 2, 200 independent X-ray confirmed DDH patients and 429 healthy controls were recruited. In total, 585 DDH patients and 987 healthy controls were recruited for this case - control candidate gene association study. DDH patients were consecutively recruited from the department of orthopedics, the first affiliated hospital of Soochow University. Controls were recruited from physical examination center in the first affiliated hospital of Soochow University. All the subjects were Han Chinese. The study was approved by the Soochow University Ethics Committee and obtained informed consents from all patients and controls. All methods were performed in accordance with the relevant guidelines and regulations.

### Sample collection

After informed consent was obtained from the families, blood samples were collected for genomic DNA extraction using the commercial kit (QIAGEN) according to the standard protocol. Hip joint capsule and ligament were obtained in 65 DDH patients during hip arthroplasty surgery. 20 age-matched trauma patients undergoing amputation surgery were recruited as control. Tissue samples were placed in separate sterile tubes and immediately stored in liquid nitrogen. Frozen samples were stored at −80 °C. Total RNA was extracted using Trizol reagent (QIAGEN) according to the manufacturer’s protocol. To avoid genomic DNA contamination in RNA, samples were treated with DNaseI (QIAGEN). Total RNA was then reverse-transcribed from 2ug of RNA using the PrimeScript RT Master Mix kit (TaKaRa).

### Genotyping of targeted locus

According to the manufacture’s protocol, the DNA of all the subjects was extracted either from the buccal swabs using the DNA IQ System (Promega, Madison, WI) or peripheral blood using the NucleoSpin Blood QuickPure Kit (Macherey-Nagel GmbH & Co. KG, Düren, German). All the samples were genotyped with Taqman assay. The sample was genotyped by uninformed laboratory personnel. Genotyping, data input and statistical results were examined by two authors independently. Five percent samples were randomly selected to repeat, and 100% consistency was obtained.

### Tissue expression of the *WIF1* gene in DDH patients and controls

The tissue expression of *WIF1* was measured with real-time PCR using gene-specific primers as follows: forward 5′-TCATGGCAGATCCAACCGTC-3′, reverse 5′-CCACTTCAAATGCTGCCACC-3′ for the *WIF1* gene, and forward 5′-GAGTC AACGGATTTGGTCGT-3′, reverse 5′-TTGATTTTGGAGGGATCTCG-3′ for the endogenous control gene Glyceraldehyde-3-phosphate dehydrogenase (*GAPDH*). For real-time PCR, 1 μL of cDNA was amplified for 40 cycles by SYBRPremix Ex TaqTM II (TaKaRa) in ABI 7900HT mentioned above. Melting curve analysis was done at the end of the reaction to assess the quality of the final PCR products. All samples were analyzed in triplicate using the 2^−ΔΔCt^ method.

### Statistics

Hardy-Weinberg equilibrium was calculated by chi-squared test in both control and case groups. The association between the DDH patients and the control subjects in the stages was tested by SAS software (version 9.2 - SAS Institute, Cary, NC, USA). Bilateral chi square tests were conducted to determine the significance of differences in allelic frequencies and P < 0.05 was considered to be statistically significant. The Student t test was used to compare the difference of WIF1 expression between the patients and the controls. DDH patients were classified into three groups according to the genotypes of each SNP, and One-way ANOVA test was used to compare the WIF1 expression among different genotypes.

## Results

### Association of rs3782499 with DDH

The distribution of genotypes in the two groups was consistent with Hardy Weinberg equilibrium (p > 0.01). In stage 1, the distribution frequency of genotype and allele of the locus rs3782499 in the Chinese Han population DDH group and the healthy controls are shown in Table 1. The results showed a significant difference in the allele frequency between the two groups (p = 0.002). There was a significant difference in genotypic distribution in the recessive pattern (AG + GG vs. AA) between the two groups (p = 0.005). A stage 2 study with independent samples was conducted to confirm the findings in stage 1. In stage 2, the significant difference in the allele frequency between the two groups was confirmed and shown in Table 1 (p = 0.008). A significant difference in genotype distribution in a recessive model (AG + GG vs. AA) between two groups (P = 0.018) was demonstrated. In total, with 585 patients and 987 controls, we demonstrated that there was a significant difference in genotype distribution in a recessive model (AG + GG vs. AA) between the two groups (p = 0.0002). The difference in the allele frequency between the two groups was shown in Table 1 (p = 4.37 * 10^−5^).

**Table 1:** Association between rs3782499 and DDH in the two-stage population.

	Genotype			Allele Frequency			P value		
	AA	AG	GG	No.	A	G	A vs G/OR(95%)^a^	AA vs others/OR(95%)	GG vs others/OR(95%)
***Stage 1***									
Case	280	100	6	386	0.86	0.15	**0.002/0.68(0.53–0.87)**	**0.005/1.50(1.13–1.99)**	**0.045/0.40(0.16–1.01)**
Control	356	181	21	558	0.80	0.20			
***Stage 2***									
Case	145	52	3	200	0.86	0.15	**0.008/0.65(0.47–0.90)**	**0.018/1.55(1.08–2.24)**	**0.063/0.33(0.10–1.12)**
Control	270	140	19	429	0.79	0.21			
***Total***									
Case	425	152	9	586	0.85	0.15	4.37*10 ^**−5**^ **/0.67(0.55–0.81)**	**0.005/1.52(1.22–1.90)**	**0.005/0.37(0.18–0.77)**
Control	626	321	40	987	0.80	0.20			

^a^OR, Odds Ratio; OR(95% confidence interval(CI)) was shown as effect allele vs reference allele. *P* values were derived from Bilateral chi square tests to determine the significance of differences.

### Tissue expression of the WIF1 in DDH and the controls

**Figure 1:**
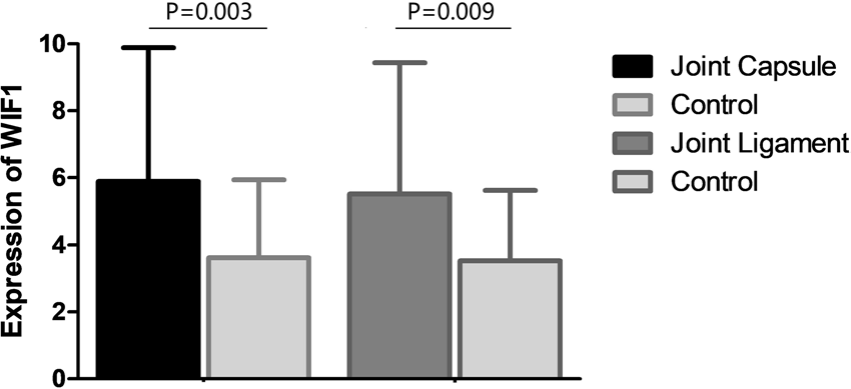
Tissue expression of WIF1 in patients and controls. DDH patients were found to have significantly higher expression of the WIF1 in the joint capsule and ligament as compared with the controls ((5.89 ± 4.36 vs. 3.62 ± 2.32, p = 0.003 for joint capsule; 5.51 ± 3.13 vs. 3.53 ± 2.25, p = 0.009 for joint ligament)).

Figure 1 summarized the expression level of the WIF1 in the patients and in the controls, respectively. DDH patients were found to have significantly higher expression of the WIF1 as compared with the controls. (5.89 ± 4.36 vs. 3.62 ± 2.32, p = 0.003 for joint capsule; 5.51 ± 3.13 vs. 3.53 ± 2.25, p = 0.009 for joint ligament).

### Relationship between the genotype of rs3782499 and the WIF1 expression

**Figure 2:**
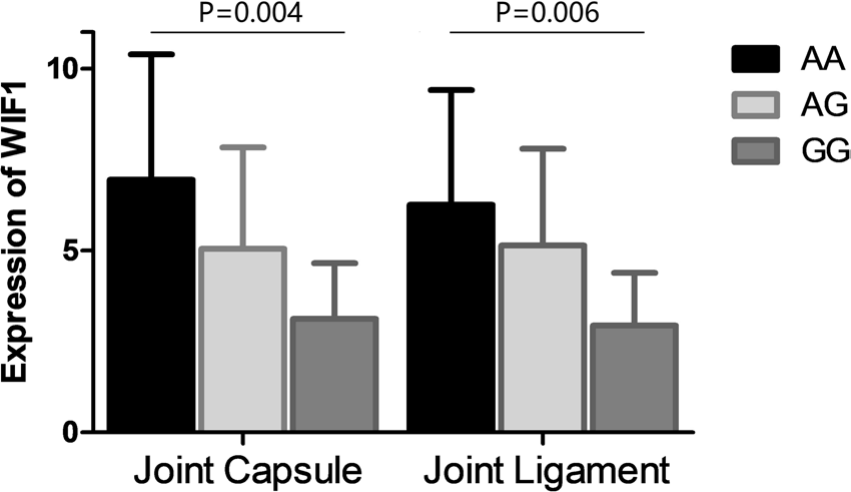
Relationship between the genotype of rs3782499 and WIF1 expression. Patients with genotype AA have a significantly increased expression of WIF1. than those with GG (*p* = 0.004 for joint capsule; *p* = 0.006 for joint ligament).

Results of the comparison of the WIF1 expression among patients with different genotypes are shown in Fig. 2. The mean value of WIF1 expression in joint capsule and joint were respectively 6.94 ± 3.45 and 6.26 ± 3.15 for genotype AA, 5.05 ± 3.12 and 5.14 ± 2.91 for genotype AG, and 3.12 ± 1.53 and 2.94 ± 1.45 for genotype GG. Patients with genotype AA were found to have a remarkably higher WIF1 expression than those with genotype GG in both joint capsule and joint ligament. (*p* = 0.004 for joint capsule; *p* = 0.006 for joint ligament).

## Discussion

In this study, we for the first time explored the association between WIF1 and DDH. A significant association between rs3782499 in gene WIF1 and DDH was identified in our two-stage study. The minor allele frequency of rs3782499 in the control group in our study (0.20) is close to that reported in HapMap for Chinese Han population (0.18). A significant difference of allele frequency between the DDH group and control group was demonstrated. Further analysis also showed significant difference in genotype distribution in a recessive model (AG + GG vs. AA) between the two groups, suggesting association between WIF1 and DDH occurrence in the Han Chinese population. We investigated the expression of WIF1 gene in hip joint capsule and ligament of DDH patients. DDH patients were found to have a significantly increased expression of WIF1 in both the capsule and ligament than the control groups. Besides, patients with genotype AA of rs3782499 were found to have a remarkably increased expression of WIF1 than those with GG. Allele A is indicative of higher expression of WIF1 as well as a higher risk of the development of DDH. Taken together, rs3782499 could be a functional variant regulating the expression of WIF1, which can further play a role in the development of DDH.

DDH is a complex multigene hereditary disease, whereby multiple susceptible genes have been reported in literature. The research conducted by Jia *et al*. showed that rs726252 in pregnancy - associated plasma protein - A2 gene was associated with DDH, suggesting a significantly greater distribution of TT genotype in DDH patients^2^. Shi *et al*. reported that ASPN was an important regulator in the etiology of DDH, reporting significant association between the D repeat polymorphism of ASPN gene and DDH^3^. In addition, Wang *et al*. found that a single nucleotide polymorphism in Tbx4 was also associated with DDH^4^ while other studies indicated GDF5 and TGFB! as susceptible genes of congenital hip dysplasia^5,6^. Furthermore, The study by Basit *et al*. indicated that there might be a functional epistatic interaction between HSPG2 and ATP2B4 in familiar DDH^7^. Basit *et al*. also identified runs of homozygosity on chromosomes 15q13.3 and 19p13.2 in a family segregating DDH with whole genome SNP genotyping^10^. All of these are suggestive of the complex genetic components in DDH.

Wnt signaling cascades are related to many stages of vertebrate limb development. They are involved in proximal-distal outgrowth and dorso-ventral patterning in the early stage and later in the development and maintenance of cartilage, bone, muscles, or joints^11^. Dysregulation of Wnt signaling has been described in several mouse lines exhibiting various limb phenotypes^12^. The classical Wnt signaling pathway is negatively regulated by a variety of soluble factors (WIF1, SFRPs and DKK) in the extracellular environment. WIF1 gene, with a unique and highly conserved WIF domain and five epidermal growth factor-like repeats, encodes a secreted protein, binding to several Wnt proteins and inhibits their activity^13^. As a secreted antagonist of WNT signaling, WIF1 is expressed mainly at the superficial layer of epiphyseal and articular cartilage and promotes chondrogenesis^14^. In a mouse arthritis model, loss of WIF1 aggravates the destruction of the cartilage^15^. Higher WIF1 expression was detected in DDH patients in the present study. We suggest WIF1 overexpression might be associated with the change of Hip cartilage content and remodeling of the macro-morphology of the hip through the suppression of Wnt signaling. Close relationship between *WIF1* gene and Wnt signaling is suggestive of a significant role of WIF1 in hip joint development. This present study was the first report concerning *WIF1* in DDH to date. Further experiment to explore the function of *WIF1* in DDH is necessary.

In conclusion, we demonstrated the polymorphism rs3782499 of *WIF1* gene was associated with DDH in Chinese Han population. Genotype AA of rs3782499 is associated with higher expression of WIF1 in DDH, which further adds to our understanding of its functional role in the development of DDH. Genetic variants of WIF1 could be a potential biomarker for the early diagnosis of DDH. Replication needs to be carried out in other ethnic populations.
